# Shaping health: conducting a community health needs assessment in culturally diverse peripheral population groups

**DOI:** 10.1186/s12939-022-01735-z

**Published:** 2022-09-12

**Authors:** Nosaiba Rayan-Gharra, Marganit Ofir-Gutler, Sivan Spitzer

**Affiliations:** Azrieli Faculty of Medicine, Bar-Ilan University, Ramat Gan, Israel

**Keywords:** Health equity, Health community needs, Periphery, Social determinants

## Abstract

**Introduction:**

The impact of social determinants on health status and outcomes has been widely established. However, it is recognized that health systems' ability to address community health needs may be limited. To better understand the interrelation between social determinants of health and health outcomes, health systems need to understand the health concerns and needs of populations. The aim of this study was to map the perceived health needs of Israel's northern periphery’s diverse ethnic and religious communities and regional clusters by conducting a community health needs assessment (CHNA).

**Methods:**

The study employed a mixed-methods approach. We conducted a CHNA in the Galilee between November 2019 to January 2020 (*n* = 750). Additionally, we conducted focus groups using design thinking methodology to better understand the underlying causes of existing gaps between community and healthcare representatives (*n* = 42). Quantitative data was analyzed using multiple logistic regressions and qualitative data was analyzed using a content and thematic analysis.

**Results:**

Galilee residents perceived sense of community (78%) as the major strength while cancer (53%) was perceived as the major health problem followed by heart disease and stroke (28.4%). The adjusted odds ratios for the association of each predictor with each perceived social and structural determinants of health among respondents indicated that Arab respondents were more likely to report race/ethnicity discrimination, domestic violence, lack of parks and recreation, neighborhood violence, limited places to exercise, school dropout and limited access to healthy food, as determinants affecting health than Jews. Conversely, Jews were more likely than Arabs to report access to mental health services, access to transportation, lack of job opportunities and access to a doctor's office as determinants affecting their health. Qualitative analysis revealed residents felt a 'lack of health security' as a result of problematic access to specialty and mental health services, especially for elderly populations.

**Conclusions:**

CHNA can inform the design of tailored interventions that will improve health for Galilee residents addressing their socioeconomic-cultural–geographical characteristics. The study's findings raise the need to create such tailored approaches to address the lack of health security felt by residents and improve not only health services provision but the social determinants affecting their health.

**Supplementary Information:**

The online version contains supplementary material available at 10.1186/s12939-022-01735-z.

## Introduction

Health and health care inequities are the result of a myriad of social, cultural, and economic factors that for the most part fall outside the traditional expertise of the health sector [1]. The impact of social determinants on health status and outcomes has been widely established [2]. However, it is also recognized that health systems' ability and capacity to address these concerns may be limited, highlighting the need for novel partnerships between health care providers and the community [3–5]. Different approaches have been implemented by health care providers around the world with the aim of addressing the social determinants of health [6]. Many interventions have stemmed from the needs of health care providers to improve effectiveness and reduce costs. For example, health care providers have addressed patient ‘no shows’ to appointments through interventions that focus on improving access to transportation [7]. Another example is the reduction of hospital readmissions of disadvantaged patients through the implementation of interventions in which social workers assist patients in addressing non-medical needs [8]. Yet, prior to their design and implementation, many interventions do not assess communities’ perceptions and needs in regards to improving access and quality of care [9].

To better understand the interrelation between social determinants of health and health outcomes, health systems need to understand populations' health concerns and needs. Both objective and perceived measures are essential in assessing communities' health needs. Perceived needs reflect how residents perceive their surrounding environment and living conditions. Objective health needs are the existing access and provision of health care services and the prevalence of disease in a given community [10]. A common way for assessing perceived health needs is by conducting Community Health Needs Assessment (CHNA, a data-driven approach to determine health needs in the service area of a health system) [11]. The Patient Protection and Affordable Care Act provision requires that nonprofit hospitals conduct CHNAs every three years so that their community’s health needs and priorities are addressed [12]. CHNA assesses community’s health needs through an interactive and dynamic process by gathering and analyzing social, economic, environmental, and health data in order to create a comprehensive profile [13]. The CHNA processes requires the direct involvement of the community, creating a unique opportunity for communities residing in social peripheries and underserved areas that are often underrepresented, and whose needs are often unheard [14]. Hence, the CHNA findings may serve as a valuable tool to connect between diverse communities' perceptions and knowledge and professional and political decision-makers, both locally and nationally [15]

The current study aimed to map the perceived health needs of residents of Israel’s northern periphery the Galilee home to 1.6 million residents by administering the first ever CHNA in Israel. Although the Israeli periphery does not meet the definition of rural or frontier, as even the most remote villages are about an hour's distance from an urban center; the Israel Central Bureau of Statistics (ICBS) defines the northern and southern areas of the country as peripheral [16]. Galilee residents have higher mortality and morbidity rates than residents living in the city of Tel Aviv or the center of Israel (the standardized to age death ratio in the Northern district is 5.3 per 1,000, whereas in Tel Aviv district, it is 4.9 per 1,000) [17, 18]. The differences between the center and the periphery are also reflected in relatively low accessibility to medical services, lower number of hospital beds and fewer specialized departments, and the longest waiting times per capita. Additional and significant gaps are apparent in medical staff distribution (1000 per person). Recent data (years 2015–2017) demonstrates that although some progress was made, the gap between the Northern district and Tel Aviv remains both in doctors (2.1 in the north and 5.3 in Tel Aviv); nurses (4.5 in the north 6.2 in Tel Aviv); as well as allied health professions (3.5 in the north, 6.8 in Tel Aviv) [19].

Despite the persisting health disparities observed, and the lack of consistent policies to reduce observed gaps in care in Israel's north periphery [19], till now a community needs assessment has not been conducted to better understand the diverse ethnic and religious or regional municipal clusters’ needs to develop interventions that may mitigate and reduce the observed health inequities [20].

## Methods

### Study 1: Quantitative study

#### Study sample and setting

The study included a convenience sample of 789 participants recruited through snowball methods. Participants who fully completed the CHNA survey were included in the final sample (*N* = 750). The CHNA data were collected from November 2019 to January 2020 in the Galilee, Israel's northern periphery. The Galilee region spans 4,473 square km, is comprised diverse cultural population of which 53% are Arab and is divided into 61 municipalities and 15 regional councils, and 17 cities. The municipal and regional councils are clustered into five municipal clusters: *Eastern Galilee*, which includes the Golan Heights; *Western Galilee* which includes the major coastal cities of Acre and Nahariya; *Beit HaKerem* which includes Karmiel, Misgav and surrounding Arab villages; *Galil Amakim* which includes the Arab town of Sakhnin and Nazareth, and *Kineret Amakim* the newest of the five municipal clusters formed in 2018 and includes 15 municipalities [21].

#### Data collection

The CHNA survey was adapted from the rural health needs assessment model [22] and tools used by University of Chicago Medicine and Johns Hopkins [23, 24] as part of their effort to meet the Patient Protection and Affordable Care Act Sect. 501(r) to the Internal Revenue Service Code [12]. The survey was translated into Hebrew and Arabic. We received 88 responses to the survey in Arabic. Seventeen Arab participants who responded in Hebrew were coded as Arab respondents (Arabs: *N* = 105 (14%)). Six hundred and forty-five participants answered the Hebrew version and were coded as Jewish respondents (Jews: *N* = 645 (86%)).

Questions asked respondents about the health status of their communities, community strengths, opportunities for improvement, and priority health needs.

The final tool included 19 questions—3 related to respondents' community of residence, 12 demographic questions, 2 multi-select questions about health problems and what they perceive is needed to create a healthy community, and 2 open-ended questions about what they perceive as strengths and health needs (See supplementary file 1: Appendix A). An electronic link to the survey was disseminated across the Galilee through emails, online platforms, community online networks and Facebook groups, local municipal communications officers, community coordinators in Kibutzim, and local community contacts and partners.

#### Quantitative measures

##### Dependent variables

The CHNA survey included two open ended questions to enable Galilee residents to express their primary health needs and what they perceive as their community's strengths. We conducted a content analysis and generated the following binary variables indicating whether each aspect was (1 = yes) or was not (0 = no) an indicated need.

- Health needs of the community: 1. Health promotion and preventive medicine; 2. Hospitals; 3. Community mental health services; 4. Emergency services 5. Childcare; 6. Elderly services; 7. Community health services; 8. Access to specialists.
- Community strengths: 1. Quality of life; 2. Social services; 3. Sense of Community.
- Additional survey items were multi-choice questions in which respondents marked relevant health problems as well as social and structural determinants affecting health (See supplementary file 1: Appendix A).
- Health problems: 16 multiple-choice items such as: cancer, dental problems, diabetes, heart disease and stroke, mental health, obesity, smoking, etc.
- Social and structural determinants of health: 18 multiple-choice items such as: limited access to healthy food, neighborhood violence, child abuse, racial/ethnic discrimination, poverty, etc.

##### Independent and control variables

- Independent variables: Ethnicity (0. Arabs; 1. Jews) and Municipal clusters (1. Eastern Galilee; 2. Western Galilee—Beit HaKerem; 3. Galil Amakim—Kineret Amakim).
- Control variables: gender (0. Males; 1. Females), age of respondent (1. 18–20; 2. 30–39; 3. 40–49; 4. 50–64; 5. 65–74; 6. 75 +), Education levels (1. High School-diploma; 2. Professional school; 3. BA; 4. MA and above), Locality (0. City; 1. Village); Religiosity (1. not religious; 2. Not so religious; 3. Religious and very religious); Number of children (continuous), and Income (continuous).

#### Statistical analysis

Descriptive statistics of respondent characteristics were calculated for the entire sample as well as according to ethnicity. In addition, we conducted a sub-group analysis to assess whether perceptions differ according to the municipal cluster of residence. Associations between the independent and control variables (respondent characteristics), and dependent variables (community strength, health needs, health problems and social determinants items) for both ethnicity and municipal clusters were assessed using chi-square tests. Multiple logistic regressions were used to measure the association between each dependent variable and the independent and control variables (see supplementary file 2). Categorical variables were recorded as dummy variables before being included in the multiple logistic regressions. In addition, the municipal clusters variable was recoded due to low response rate in two clusters. Clusters were merged together according to geographic adjacency. Western Galilee was merged with Beit-HaKerem, and the Galil Amakim with Kineret Amakim. Findings reported for the univariate analyses include any variable found to have a *p* value < 0.05, however, a Bonferroni correction for multiple testing removes statistical significance, except where *p* < 0.0012. Analyses were performed using IBM SPSS Statistical package V.27.0.

Ethics approval for the study was obtained from the ethical review board of the Azrieli Faculty of Medicine.

### Study 2: Qualitative study

#### Participants and procedure

Following the CHNA survey, we conducted focus groups to get a better understanding of the underlying causes of existing gaps between community and healthcare representatives [22]. We identified stakeholders across the Galilee representing either health care system organizations, i.e., Ministry of Health’s Northern district (*n* = 2), the four national Health Maintenance Organizations (HMOs: *n* = 15) and regional hospitals (*n* = 5), or communities which were represented by municipal (*n* = 13), local NGOs (3) and patients (*n* = 4). We invited them to participate in a workshop. Fifty participants agreed to participate, of which 42 attended the workshop.

Participants were divided into 10 pre-determined groups according to the five geographic clusters and affiliations (health care system (*n* = 21) or community (*n* = 21)). Using Design Thinking methodology [25], each group first worked on defining the problem by addressing the goals, pains and gains from their perspective. Following this session, the two groups in each cluster, health system and community, met together, shared their maps, and then worked together to align needs and ideate possible collaborations and solutions.

#### Qualitative data analysis

We conducted a content analysis of qualitative data to identify significant themes. Discussion of workshop participants as well as the maps participants filled out were summarized and analyzed deriving major themes.

## Quantitative results

### CHNA survey

A total of 750 respondents completed the survey, 645 of those were in Hebrew and 105 responses to the survey in Arabic. Our survey included 424 (56.5%) respondents from the Eastern Galilee, 165 (22%) respondents from the Western Galilee- Beit Hakerem, 161 (21.5%) respondents from Galil and Kineret Amakim cluster.

Respondents were predominantly female (77.3%), Jewish (86%), secular (66.8%) with academic diplomas (73.9%), and middle class according to their reported monthly household income (13,000–17,000 NIS (22.3%), 17,001 – 24,000 NIS (20.8%)). About 53% of respondents' age ranged from 40–64, and on average, they had three kids. 78.3% of the respondents lived in a village (Table 1).

**Table 1:** Sample characteristics for the entire sample and by Ethnicity

	**Total** **N (%)**	**Jewish *N* = 645**	**Arab** ***N* = 105**	***P*. Value**
**Sex (%female)**	580 (77.3)	514 (79.7)	66 (62.9)	***P* < 0.001**
**Age**				
18–29	57 (7.6)	**37 (5.7)**	**20 (19)**	***P* < 0.001**
30–39	180 (24)	149 (23.1)	31 (29.5)	
40–49	200 (26.7)	166 (25.7)	34 (32.4)	
50–64	196 (26.1)	**179 (27.8)**	**17 (16.2)**	
65–74	99 (13.2)	**96 (14.9)**	**3 (2.9)**	
+ 75	18 (2.4)	18 (2.4)	0	
**Religion**				
Jewish	645 (86)	645 (100)	0	***P* < 0.001**
Christian	47 (6.3)	0	47 (44.8)	
Muslim	42 (5.6)	0	42 (40)	
Druze	16 (2.1)	0	16 (15.2)	
**Religiosity**				
Not religious	501 (66.8)	**458 (71)**	**43 (41)**	***P* < 0.001**
Not so religious	100 (13.3)	**62 (9.6)**	**38 (36.2)**	
Religious	98 (13.1)	85 (13.2)	13 (12.4)	
Very Religious	28 (3.7)	26 (4)	2 (1.9)	
Don't know	23 (3.1)	**14 (2.2)**	**9 (8.6)**	
**Education**				
High School diploma	100 (13.3)	91 (14.1)	9 (8.6)	***P* = 0.021**
Professional school	96 (12.8)	**90 (14)**	**6 (5.7)**	
BA	302 (40.3)	**250 (38.8)**	**52 (49.5)**	
MA and above	252 (33.6)	214 (33.2)	38 (36.2)	
**Household Monthly Income**				
Less than 2500 NIS	13 (1.8)	11 (1.8)	2 (2)	*P* = 0.353
2,501–4,000 NIS	17 (2.4)	12 (2)	5 (4.9)	
4,001–5,000 NIS	20 (2.8)	16 (2.6)	4 (3.9)	
5,001–6,500 NIS	27 (3.8)	21 (3.5)	6 (5.9)	
6,501 – 8,000 NIS	58 (8.2)	49 (8.1)	9 (8.8)	
8,001—10,000 NIS	80 (11.3)	71 (11.7)	9 (8.8)	
10,001 – 13,000 NIS	87 (12.3)	79 (13)	8 (7.8)	
13,001 – 17,000 NIS	158 (22.3)	140 (23)	18 (17.6)	
17,001 – 24,000 NIS	148 (20.8)	122 (20.1)	26 (25.5)	
Over 24,001 NIS	102 (14.4)	87 (14.3)	15 (14.7)	
**Municipal clusters**				
Eastern Galilee	424 (56.5)	**410 (63.6)**	**14 (13.3)**	***P* < 0.001**
Western Galilee – Beit HaKerem	165 (22)	**153 (23.7)**	**12 (11.4)**	
Galil Amakim—Kineret Amakim	161 (21.5)	**82 (12.7)**	**79 (75.2)**	
**Locality**				
City	163 (21.7)	138 (21.4)	25 (23.8)	*P* = 0.578
Village	587 (78.3)	507 (78.6)	80 (76.2)	
**Perceived health status**				
Not good	63 (8.5)	59 (9.2)	4 (3.8)	***P* = 0.001**
Good	325 (43.6)	**291 (45.5)**	**34 (32.4)**	
Very good	357 (47.9)	**290 (45.3)**	**67 (63.8)**	
**Children number (Mean ± SD)**	2.64 (1.48)	2.77 (1.47)	2.0 (1.41)	***P*** **< 0.001**

Both Arabs and Jewish respondents perceived themselves to be in 'good' or 'very good' health (43.6% and 47.9%, accordingly). Overall, we found significant differences in the characteristics of Jewish in comparison to Arab respondents (Table 1). When we compared groups characteristics according to their residence in municipal clusters, minimal differences were found, alluding to a regional socio-demographic similarity between the clusters (data not shown).

### Community strengths

Sense of community such as solidarity, caring for one another, and the knowledge that someone will help in a time of need was deemed the main strength of the Galilee communities (78.1%). Interestingly, “quality of life” (30.3%) such as open spaces, good neighbours, clean air, nice view, and quiet place, and “community services” such as social support, cultural activities, and activities for older adults and children (22.9%) were not perceived as a major strength. When comparing Arab and Jewish respondents on perceived community strengths, we found significant differences (Table 2). Jewish respondents rated a sense of community highly (81.7%), as opposed to Arab respondents (55.2%); Quality of life was rated by almost a third of the Jewish respondent as a strength, whereas only 14.3% of Arab respondents identified it as such.

**Table 2:** Community Health Needs Assessment survey by Ethnicity

	**Total** ***N* = 750**	**Jewish *N* = 645** **(86%)**	**Arab *N* = 105** **(14%)**	***P*. value**
**Community strengths** (%yes)				
Quality of life	230 (30.3)	211 (32.7)	15 (14.3)	***P* < 0.001**
Sense of community	593 (78.1)	527 (81.7)	58 (55.2)	***P* < 0.001**
Community services	174 (22.9)	156 (24.2)	18 (17.1)	*P* = 0.11
**Health needs of the community** (%yes)				
Health promotion and preventive medicine	110 (14.5)	87 (13.5)	22 (21)	***P* = 0.04**
Hospitals	89 (11.70)	75 (11.6)	14 (13.3)	*P* = 0.62
Community mental health services	19 (2.50)	18 (2.8)	1 (1)	*P* = 0.27
Emergency services	135 (17.8)	122 (18.9)	10 (9.5)	***P* = 0.02**
Childcare	119 (15.70)	98 (15.2)	20 (19)	*P* = 0.32
Elderly services	128 (16.90)	107 (16.6)	18 (17.1)	*P* = 0.88
Community health services	361 (47.60)	306 (47.4)	53 (50.5)	*P* = 0.56
Access to specialists	251 (33.1)	209 (32.4)	39 (37.1)	*P* = 0.34
**Health problems** (%yes)				
Age-related illness	322 (42.9)	305 (47.3)	17 (16.2)	***P* < 0.001**
Cancer	400 (53.3)	339 (52.6)	61 (58.1)	*P* = 0.29
Dental problems	130 (17.3)	119 (18.4)	11 (10.5)	***P* = 0.045**
Diabetes	207 (27.6)	154 (23.9)	53 (50.5)	***P* < 0.001**
Heart disease and stroke	213 (28.4)	176 (27.3)	37 (35.2)	*P* = 0.094
Infectious diseases	63 (8.4)	62 (9.6)	1 (1)	***P* = 0.003**
Lung disease (COPD)	36 (4.8)	33 (5.1)	3 (2.9)	*P* = 0.32
Mental Health	138 (18.4)	126 (19.5)	12 (11.4)	***P* = 0.047**
Mother and Infant Health	227 (30.3)	221 (34.3)	6 (5.7)	***P* < 0.001**
Motor and Vehicle Crash	68 (9.1)	47 (7.3)	21 (20)	***P* < 0.001**
Obesity	145 (19.3)	110 (17.1)	35 (33.3)	***P* < 0.001**
Smoking	72 (9.6)	51 (7.9)	21 (20)	***P* < 0.001**
Sexual Transmitted Infections	2 (0.3)	2 (0.3)	0	*P* = 0.568
Substance Abuse	37 (4.9)	28 (4.3)	9 (8.6)	*P* = 0.06
Violence	30 (4.0)	4 (0.6)	26 (24.8)	***P* < 0.001**
Other	6 (0.9)	6 (0.9)	0	*P* = 0.321
Don't know	19 (2.6)	19 (2.9)	0	*P* = 0.216
**Social and structural determinants of health** (%yes)				
Residence near a polluting factory	45 (6.0)	34 (5.3)	11 (10.5)	***P* = 0.037**
Race/ethnicity discrimination	18 (2.4)	6 (0.9)	12 (11.4)	***P* < 0.001**
Pollution	50 (6.6)	37 (5.7)	13 (12.4)	***P* = 0.011**
Access to mental health services	185 (24.6)	178 (27.6)	7 (6.7)	***P* < 0.001**
Domestic violence	14 (1.8)	4 (0.6)	10 (9.5)	***P* < 0.001**
Access to transportation	274 (36.5)	268 (41.6)	6 (5.7)	***P* < 0.001**
Poverty	40 (5.3)	31 (4.8)	9 (8.6)	*P* = 0.111
Affordable housing	43 (5.8)	30 (4.7)	13 (12.4)	***P* = 0.002**
Child abuse/neglect	16 (2.1)	6 (0.9)	10 (9.5)	***P* < 0.001**
Affordable childcare	103 (13.7)	79 (12.2)	24 (22.9)	***P* = 0.003**
Parks and recreation	160 (21.3)	97 (15)	63 (60)	***P* < 0.001**
Neighborhood safety/violence	49 (6.5)	16 (2.5)	33 (31.4)	***P* < 0.001**
Limited places to exercise	173 (22.9)	136 (21.1)	37 (35.2)	***P* = 0.001**
Lack of job opportunities	246 (32.8)	237 (36.7)	9 (8.6)	***P* < 0.001**
School dropout/poor schools	40 (5.3)	23 (3.6)	17 (16.2)	***P* < 0.001**
Limited access to healthy food	92 (12.3)	71 (11)	21 (20)	***P* = 0.009**
Access to a doctor's office	445 (59.3)	436 (67.4)	9 (8.6)	***P* < 0.001**
Other	54 (7.2)	52 (9.9)	2 (1.9)	***P* = 0.024**
Don't know	7 (0.9)	7 (1.1)	0	*P* = 0.283

Table 3 demonstrates the similarities and differences according to residence in municipal clusters. Respondents residing in the Western Galilee—Beit HaKerem found the quality of life as a community strength (36.5%), whereas only 23% of respondents from Galil-Kineret Amakim identified it as a strength. Sense of community was identified by most Western and Eastern Galilee residence as a community strength (84.4% and 81.2% accordingly), and by about two third of Galil Amakim residence (63.4%).

**Table 3:** Community health needs assessment survey by municipal clusters

	**Western Galilee—Beit HaKerem *N* = 167 (22%)**	**Eastern Galilee** ***N* = 431** **(56.8%)**	**Galil Amakim- Kineret Amakim** ***N* = 161** **(21.2%)**	***P*. value**
**Community strengths** (%yes)				
Quality of life	**61 (36.5)**	132 (30.6)	**37 (23)**	***P* = 0.028**
Sense of community	141 (84.4)	350 (81.2)	**102 (63.4)**	***P* < 0.001**
Social services	42 (25.1)	97 (22.5)	35 (21.7)	*P* = 0.729
**Health needs of the community** (%yes)				
Health promotion and preventive medicine	**36 (21.6)**	**49 (11.4)**	25 (15.5)	***P* = 0.006**
Hospitals	22 (13.2)	55 (12.8)	12 (7.5)	*P* = 0.163
Community mental health services	5 (3)	10 (2.3)	4 (2.5)	*P* = 0.894
Emergency services	32 (19.2)	80 (18.6)	23 (14.3)	*P* = 0.418
Childcare	21 (12.6)	64 (14.8)	34 (21.1)	*P* = 0.08
Elderly services	20 (12)	79 (18.3)	29 (18)	*P* = 0.161
Community health services	71 (42.5)	208 (48.3)	82 (50.9)	*P* = 0.283
Access to specialists	53 (31.7)	136 (31.6)	62 (38.5)	*P* = 0.225
**Health problems** (%yes)				
Age-related illness	**80 (47.9)**	192 (44.5)	**56 (34.8)**	***P* = 0.041**
Cancer	89 (53.3)	231 (53.6)	83 (51.6)	*P* = 0.905
Dental problems	21 (12.6)	**88 (20.4)**	**23 (14.3)**	***P* = 0.038**
Diabetes	53 (31.7)	106 (24.6)	49 (30.4)	*P* = 0.133
Heart disease and stroke	**32 (19.2)**	**135 (31.3)**	48 (29.8)	*P* = 0.011
Infectious diseases	8 (4.8)	48 (11.1)	8 (5)	***P* = 0.009**
Lung disease (COPD)	11 (6.6)	23 (5.3)	3 (1.9)	*P* = 0.111
Mental Health	35 (21)	76 (17.6)	29 (18)	*P* = 0.634
Mother and Infant Health	43 (25.7)	**156 (36.2)**	31 (19.3)	***P* < 0.001**
Motor and Vehicle Crash	19 (11.4)	**26 (6)**	**23 (14.3)**	***P* = 0.003**
Obesity	33 (19.8)	**65 (15.1)**	**47 (29.2)**	***P* = 0.001**
Smoking	**11 (6.6)**	34 (7.9)	**28 (17.4)**	***P* = 0.001**
Sexual Transmitted Infections	0	1 (0.2)	1 (0.6)	*P* = 0.538
Substance Abuse	**15 (9)**	**10 (2.3)**	12 (7.5)	*P* = 0.001
Violence	0	3 (0.7)	**27 (16.8)**	***P* < 0.001**
Other	1 (0.6)	6 (1.4)	0 (2.5)	*P* = 0.225
Don't know	8 (4.8)	9 (2.1)	3 (1.9)	*P* = 0.142
**Social and structural determinants of health** (%yes)				
Residence near a polluting factory	24 (14.4)	9 (2.1)	13 (8.1)	***P* < 0.001**
Race/ethnicity discrimination	**0**	8 (1.9)	**10 (6.2)**	***P* = 0.001**
Pollution	17 (10.2)	**14 (3.2)**	19 (11.8)	***P* < 0.001**
Access to mental health services	35 (21)	**126 (29.2)**	**26 (16.1)**	***P* = 0.002**
Domestic violence	1 (0.6)	4 (0.9)	**9 (5.6)**	***P* < 0.001**
Access to transportation	**76 (45.5)**	165 (38.3)	**36 (22.4)**	***P* < 0.001**
Poverty	5 (3)	25 (5.8)	10 (6.2)	*P* = 0.323
Affordable housing	14 (8.4)	**17 (3.9)**	**13 (8.1)**	*P* = 0.043
Child abuse/neglect	2 (1.2)	8 (1.9)	7 (4.3)	*P* = 0.112
Affordable childcare	27 (16.2)	50 (11.6)	28 (17.4)	*P* = 0.118
Parks and recreation	**24 (14.4)**	74 (17.2)	**64 (39.8)**	***P* < 0.001**
Neighborhood safety/violence	5 (3)	4 (0.9)	**40 (24.8)**	***P* < 0.001**
Limited places to exercise	30 (18)	103 (23.9)	41 (25.5)	*P* = 0.208
Lack of job opportunities	45 (26.9)	**174 (40.4)**	30 (18.6)	***P* < 0.001**
School dropout/poor schools	12 (7.2)	**12 (2.8)**	16 (9.9)	***P* = 0.001**
Limited access to healthy food	24 (14.4)	50 (11.6)	20 (12.4)	*P* = 0.653
Access to a doctor's office	**95 (56.9)**	**303 (70.3)**	**54 (33.5)**	***P* < 0.001**
Other	14 (8.4)	36 (8.4)	5 (3.1)	*P* = 0.074
Don't know	1 (0.6)	4 (0.9)	2 (1.2)	*P* = 0.830

The adjusted odds ratios for the association of the independent variables with each *perceived community strength measure* (controlling for the community characteristics- control variables) are shown in Table 1s (supplementary file 2). Non-religious respondents, compared to religious respondents, were more likely to report community services as a strength. Jewish respondents were more likely than Arabs to report quality of life and sense of community as strengths. Sense of community was also more likely to be reported by respondents who live in a village and have an academic degree as opposed to those residing in the city and/or non-academic (*p* < 0.0012).

### Health needs assessment of the community

When we asked respondents to openly reply and answer what they think are the major health needs of their community, we found two main domains (Table 2): the lack of basic Health services in the community (47.6%) and problematic access to specialists, including long waiting times or the need to travel great distances to receive care (33.1%). In addition, emergency services, and age-based services such as the lack of professional services for the elderly in the region, including availability of gerontologists were also found to be a major need (17.8%, 16.9%, accordingly).

Health Promotion and preventive medicine were deemed by 14.5% of respondents as an important need. We found a significant difference between Arab and Jewish respondents, with about a fifth (21%) of Arab respondent as opposed to only 13.5% Jews mentioning the lack of health promotion infrastructure (Table 2) such as outdoor sport equipment, walking/running/cycling trails, public parks, and the existence of environmental hazards such as polluting factories in their community. Additionally, many Arab respondents mentioned the lack of financial support for health promoting activities such as afterschool activities/sport activities.

Interestingly, health promotion and preventive medicine was the only need that was significantly different according to municipal cluster of residence, with respondents in the Western Galilee—Beit HaKerem (21%) stating it as a major issue as opposed to those residing in the Eastern Galilee (11.4%) (Table 3).

Table 2s (supplementary file 2) presents adjusted odds ratios for the association of the independent variables with each *community health need*. After adjusting for all respondents' characteristics, all the outcomes of the perceived community health needs were not found to be significant (*p* > 0.0012).

### Identified health problems

When assessing needs according to respondents' ethnicity, we found significant differences in the awareness and perceptions of health problems. Univariate analysis showed that both Jews and Arabs perceived cancer (53%) as the major health problem followed by heart disease and stroke (28.4%). Besides cancer and cardiovascular disease, Jews identified additional major health problems to be age-related Illnesses such as arthritis, vision/hearing loss, dementia (47.3%), and mother and Infant health (34.3%). In comparison, Arab respondents identified additional major health problems to be: diabetes (50.5%), obesity (33.3%) and violence (24.8%) (Table 2).

Health problems were found to be significantly different according to the clusters of residence, with *age-related illness* identified by 47.9% of the 'Western Galilee—Beit HaKerem" as a major issue; Eastern Galilee respondents identified *heart disease and stroke* (31.3%) and *mother and infant health* (36.2%) as their major health problems; *Obesity* was identified as a health problem by "Galil Amakim- Kineret respondents" (29.2%); *Smoking* was identified by 17.4% of "Galil Amakim- Kineret Amakim" respondents. Finally, v*iolence* was identified as a health problem by 16.8% of "Galil Amakim- Kineret Amakim" respondents (Table 3)**.**

Table 3s (supplementary file 2) presents adjusted odds ratios for the association of the independent variables with each *perceived community health problem***.** After controlling for respondents' characteristics, ethnic differences remained significant, with Jewish respondents more likely to report age-related illness and mother and Infant health than Arab respondents. Arabs were more likely than Jews to report diabetes, obesity, and violence as health problems.

### Social and structural determinants of health

Identification of structural social determinants and their effect on community health differed significantly between Arabs and Jews in all aspects (Table 2), apart from poverty, which was shared by both communities. Access to a doctor's office (67.4%), access to transportation (41.6%), and lack of job opportunities (36.7%) were the three most important barriers perceived by Jews. Access to mental health services was also found as an essential barrier (27.6%). Survey findings in Arabic reflect a different picture in which parks and limited places to exercise (60%, 35.2% accordingly), neighborhood safety (31.4%), and affordable childcare (22.9%) were perceived as the structural social determinants impeding communities' health (Table 2).

Respondents from "*Galil Amakim- Kineret Amakim"* significantly identified Parks and recreation (39.8%), access to a doctor's office (33.5%), neighborhood safety/violence (24.8%), access to transportation (22.4%), and access to mental health services (16.1%) as the most important social and structural determinants of health. Interestingly, respondents from the *Eastern Galilee* cluster identified access to a doctor's office (70.3%), lack of job opportunities (40.4%), and access to mental health services (29.2%) as the main social and structural determinants of health. Similar to the eastern Galilee cluster, residents in the *Western Galilee—Beit HaKerem* clusters identified access to a doctor's office (56.9%) as an important social structural determinant, but in addition stated that access to transportation as important (45.5%), and to a lesser extent parks and recreation (14.4%) (Table 3).

Tables 4s and 4.1s (supplementary file 2) present adjusted odds ratios for the association of the independent variables with each *perceived Social and structural determinant of the health* of the communities controlling for the respondents' characteristics. *Arab respondents* were more likely to report race/ethnicity discrimination, domestic violence, child abuse/neglect, affordable childcare, lack of parks and recreation, neighborhood violence, limited places to exercise, school dropout/poor schools, and limited access to healthy food, as determinants affecting health than Jews. Conversely, *Jews* were more likely than *Arabs* to report access to mental health services, access to transportation, lack of job opportunities and access to a doctor's office as determinants affecting their health.

Respondents from *Western Galilee – Beit HaKerem* clusters were more likely to report residence near a polluting factory; respondents from *Galil Amakim – Kineret Amakim* were more likely to report neighborhood violence than Eastern Galilee. In addition, respondents who live in a* village* were more likely to report access to transportation and access to a doctor's office as determinants affecting health than respondents who live in a city. In comparison, respondents who live in the *city* were more likely to report a lack of parks and recreation and neighborhood violence as social determinants of health.

### Qualitative results

#### The community-health services interface – identifying key issues and possible solutions from the focus groups

In accordance with the rural health needs assessment model [22] and following the CHNA analysis, we conducted focus groups to better understand Galilee communities' pains and gains. Focus groups participants, both community and health services representatives, raised three main 'pains' in current health care provision: Low awareness of the population to the importance of prevention and lack of supporting policies to do so; quality of care provided; lack of infrastructure.

- **1. Prevention**—Low awareness and lack of supporting policies: Participants in all focus groups stressed the low awareness of the Galilee population to the importance of maintaining a healthy lifestyle and preventing chronic disease. This was often paired with the lack of policies and infrastructure to create a support system to promote such activities including: Lack of public spaces for exercise; Low access to healthy foods due to high cost and low availability; Lack of nutritional services that are culturally fit and affordable.
- **2. Quality of Care** – Barriers to quality care were mentioned both from the provider and patient perspectives. Barriers in access to professional training and low awareness of clinicians to new clinical recommendations, technologies, and drugs. Lack in provision of tailored services to fit the community served, such as the lack of appropriately tailored dietary counseling to Arab as well as ultraorthodox populations. Lack of awareness to the effects of socio-economic determinants in treatment plans, such as prescribing medications and treatments that the patients cannot afford Lack of integration among care providers, a case manager of sorts, to help mitigate the sense of 'being lost' those patients and their families feel while trying to navigate the system.
- **3. Lack of infrastructure**—both patients and providers talked of the effect the problematic Galilee infrastructure and lack of budget have on access to and quality of care. Not enough specialists in the periphery; Lack of specialist clinics, such as foot clinics and distance and problematic public transportation make access incredibly difficult.

It is important to note that the community representatives differed in their 'pain perspectives'. While municipal representatives knew the overall macro burdens regarding access to care, they lacked a deeper understanding of the barriers affecting patients that NGOs representatives expressed. Interestingly, we did not find a difference, but rather a consensus in the 'pains' perceived by community and health care representatives across the five municipal Galilee clusters.

The discussion on gains was dominated by the health system representatives, who shared the many activities, interventions and services provided to improve care. These included patient education activities, community volunteering to improve prevention awareness, technological innovations such as development of new apps or remote care infrastructure.

### Potential solutions and collaborations

Both system and community representatives worked together to ascertain possible solutions and collaborations to improve care in the region. We identified in the five municipal clusters three common over-arching areas for intervention and improvement: Improvement of care provision, community-based partnerships to improvement prevention awareness, and institutional integrated care model to provide quality care.

- **1. Improving the quality of care** – interventions included: Creating an infrastructure for patient-family-provider partnership to improve care; Professional training not only on clinical but cultural and community focused practices; Provision of remote accessible services such as 24/7 pharmaceutical service; Addressing the psychological and not only the clinical aspects of chronic disease as part of treatment; Creating system support for assisting disadvantaged patients in acquiring medications, healthy foods, and exercise.
- **2. Community-based prevention partnerships** – the community representatives mentioned that often, they are interested in setting-up such partnerships, but do not always have the skills or tools to do so. Solutions suggested included: A 'Health Promotion and Prevention Van' that would travel between different communities with relevant educational materials; Creating a community wellness infrastructure for at risk patients such as subsidized memberships to the gym; Creating community-health system-education system partnerships to address prevention from an early age and creating strong voluntary health promotion counselors made up of retired health professionals that reside in the region.
- **3. Creating a regional integrated care model** – bringing together third and private sectors, municipalities, and the healthcare system to create an integrated care model to address the care pathway from prevention to care. This includes building a complex multi-faceted model in which all partners work together and invest resources. While there were small nuances in the model suggested, such as the place of religious leaders within the care-provision model, all groups believed that creating a holistic care model is essential for the region.

## Discussion

We conducted the first-ever community health needs assessment (CHNA) in the Galilee region. Our main goals were to identify the needs, problems, and strengths as the diverse Galilee communities perceived them. As Marmot stated, empowering individuals and communities is key to reducing health inequities and promoting populations' health [26–28]. The findings of this CHNA can serve as a basis for designing and implementing interventions to improve not only the living conditions but reduce health inequities for Galilee residents so that they may live flourishing lives regardless of their place of residence, gender, ethnicity, and socio-economic status.

Our multivariate analysis findings show that municipal clusters were not significantly different in their health needs and perceptions of social determinants. As such, the Galilee region can be perceived as a fairly homogenous geographical area. Common health needs included health promotion and preventive medicine, access to community health services and specialists, and chronic diseases (cancer, diabetes, and obesity). These findings were also supported by focus groups participants that highlighted the low awareness and lack of supporting policies for health prevention. They also stated that the main barriers to attaining quality health care such include access to technology and professional training as well as the lack of providers' awareness to tailoring services that address the diverse communities' characteristics and needs alongside the lack of infrastructure and access to community health services and specialists.

Interestingly, ethnicity was the major driver for differing perceptions of health problems. While common issues of health promotion and preventive medicine were shared by both Arab and Jewish respondents, Arabs significantly perceived diabetes, obesity, and violence as the most pressing health problems. Both Jewish and Arab respondents identified social and structural problems such as residence near a polluting factory, poverty, and the need for affordable housing as concerns. However, Arabs reported more social problems than Jews, such as ethnic discrimination, domestic violence, child abuse, affordable childcare, lack of parks and recreation, neighborhood violence, a place to exercise, school dropout, and limited access to healthy food.

The differences observed in our survey between Arab and Jewish communities, albeit similarities attributed to residing in the same regional unit, demonstrate the "double periphery" phenomenon [29]. The Arab population residing in the north of the country suffers not only from residing in the geographic periphery but also from peripheral social status due to their ethnicity. The double periphery analytical and theoretical framework views peripherality as the politicization of remoteness, distinctiveness, and dependence, combined with the peripherality of minorities [30, 31]. A recent report published by the Taub Center, comparing between the Arab and Jewish communities in Israel, found that Arabs are less likely to use health services and primarily specialists [32]. In addition, Arabs are more likely to use health services when their health becomes severe, a pattern of behavior that characterizes lower-income communities and can be influenced by social and cultural characteristics [33]. These findings coincide with the annual inequality reports published by the Israeli Ministry of Health in which an ongoing gap between Israel's peripheries and center districts regarding health services use and access, both in the community and hospitals setting as well as shortage of professional medical staff persist [34, 35]. When tailoring solutions, one should address the Northern region's geographical similarities, but bear in mind the ethnicity-based differences that require equitable resource allocation for the Arab society as well as culturally competent social and health services.

Despite an array of perceived health needs and challenges, sense of community was perceived as a strength by all clusters, regardless of ethnicity, and was seen as an added value for living in the Galilee. It has been recognized that poor health found in deprived communities is a result not only of low socio-economic status but also low sense of community [36, 37]. Improving the sense of community through social activities, integration and communication can offset an individual and community's wellbeing and reducing the harmful health effects of social isolation [38–40]. Consequently, a recent study demonstrated that strengthening disadvantaged communities by empowering their sense of place and community as well as creating partnerships between community groups, services providers, health commissioners, and academia may assist in addressing societal and structural health inequities [41].

CHNA surveys may be a useful tool for promoting sense of community and addressing structural inequities by bringing to light needs of the periphery alongside downstream policies. Civil society organizations and academia have long examined the state of health in the peripheries and offered alternative solutions to current health system models [19]. One such example is the work done by Northern Ontario School of Medicine (NOSM). NOSM, located in the underserviced rural communities of northern Ontario, engaged these communities and identified their need to develop a health workforce that is sustainable and responsive to community needs [42, 43]. Similarly, the Azrieli Faculty of Medicine, Bar-Ilan University operates out of and within the northern periphery of Israel. As such, it views itself as a partner in developing upstream processes, by mapping and identifying needs as well as developing and implementing interventions. We believe that enabling communities to become active players in shaping their lives and responding to their perceived needs will strengthen their trust in local institutions. The CHNA we conducted led to the development of a new initiative: The Russel Berrie Galilee Diabetes SPHERE launched November 2021. SPHERE promotes partnerships between academia, local authorities, HMOs, hospitals, and civil society, on a regional basis to reduce structural barriers and diabetes disparities. Taking into account the CHNA identified unique needs of each community, SPHERE is driven by the concept of health equity, in contrast to the concept of health equality promoted by government ministries [44], designing tailored interventions to address cultural, ethnic, health literacy, geographical and planning characteristics.

### Limitations

There are a number of limitations to the snowball methodology we adopted. Our sample is lacking in representation of different Galilee communities, mainly Arab speaking and Jewish Ultraorthodox communities. As this survey was disseminated electronically, it was difficult to reach ultraorthodox residents who do not use electronic interface or social media [45]. Additionally, as can be seen, we encountered low response rates among the Arab sector. We consulted local experts from the Galilee Society Research Institute, which conducts the largest Arab household survey in the country, to better understand this phenomenon [46] Interviews we conducted with the director and head of research of the Galilee Society highlighted that low response rate to surveys conducted in Arabic is not uncommon in Israel. Lack of trust and political climate were described as major barriers in conducting surveys in the Arab population [47]. To overcome this and encourage response and trust, we asked for assistance in dissemination through key stakeholders in the Arab Galilee ecosystem, such as the Galilee Society Research Institute, Faculty members, and leading municipal leaders. Additionally, we strategically sought out the participation of representatives from the Arab and Jewish ultraorthodox communities in the focus groups conducted. The analysis did not reveal significant different perceptions than those found in the CHNA survey.

## Conclusions

This study highlights the diverse needs of residents residing in social-geographical peripheries. Among Galilee residents, for example, the Arab community perceived more structural and social inequities than Jewish residents. Our findings raise the need to create diverse responses both in terms of health services and social determinants. To address the concerns and needs of peripheral populations a possible suggested solution is to use existing community resources and increase accessibility and quality of care through integration of services of the different health and community services providers. Implementing such interventions require tailoring to residents socioeconomic-cultural–geographical characteristics, providing ultimately not only health security to all, but reducing the persisting health inequities.

## Supplementary Information

**Additional file 1: Appendix A.** Community health needs assessment.

**Additional file 2: Table 1s.** Adjusted odds ratios from multivariate logistic regression models predicting perceived community strengths among respondents (*N* = 759), Galilee residents Israel. **Table 2s.** Adjusted odds ratios from multivariate logistic regression models predicting perceived health needs among respondents (*N* = 759), Galilee residents Israel. **Table 3s.** Adjusted odds ratios from multivariate logistic regression models predicting perceived health problems among respondents (*N* = 759), Galilee residents Israel. **Table 4s.** Adjusted odds ratios from multivariate logistic regression models predicting social and structural determinants of health among respondents (*N* = 759), Galilee residents Israel. **Table 4.1s.** Adjusted odds ratios from multivariate logistic regression models predicting social and structural determinants of health among respondents (*N* = 759), Galilee residents Israel.

## Data Availability

Not applicable.
