# Supplementary material for: Shaping health: conducting a community health needs assessment in culturally diverse peripheral population groups
Source: Int J Equity Health. 2022 Sep 12;21:131. doi: 10.1186/s12939-022-01735-z (PMC9469555; doi:10.1186/s12939-022-01735-z)
Supplement: Supplementary file 1 — Additional file 1: Appendix A. Community health needs assessment. [file 12939_2022_1735_MOESM1_ESM.pdf]

Supplementary file 1: Appendix A

Community Health Needs Assessment

The BIU Azrieli Faculty of Medicine is conducting a survey to map the health needs of the Galilee residents. It's important for us to understand what is important to you, what are your personal health needs as well as your community's. Your input will help us not only understand your community's needs but also to design a strategy for action.

The survey should take only 5 minutes to complete. Your responses are anonymous, and you will not be asked your name.

*You must be 18 years or older to complete this survey.

If you have any questions about the survey - please contact Dr. Sivan Spitzer-Shohat from the Department of Populaiton Health, Azrieli Faculty of Medicine, Bar-Ilan University by email: sivan.spitzer-shohat@biu.ac.il or phone: 072-2644907.

Thank you for your cooperation!

7.1.1 Tell us about your community and the area you live in:

Where do you live?

What neighborhood or community do you live in?

What are the greatest strengths or best things in the community where you live in? (List up to 3)

What do you think are the health / wellbeing needs of the community where you live in?

What do you think are the three most important health problems effecting your community? (Choose 3)

- ☐ Age-related illness (arthritis, hearing/vision loss, Alzheimer's/dementia, etc.)
- ☐ Cancer
- ☐ Dental problems
- ☐ Diabetes (high blood sugar)
- ☐ Heart disease and stroke
- ☐ Infectious diseases (hepatitis, TB, flu, etc.)
- ☐ Lung disease (asthma, COPD, etc.)
- ☐ Mental health (depression, anxiety, PTSD, suicide, etc.)
- ☐ Mother and infant care
- ☐ Motor vehicle crash injuries
- ☐ Obesity
- ☐ Smoking
- ☐ Sexually Transmitted Infections (STIs/STDs) including HIV
- ☐ Substance-use (alcohol, prescription misuse and other drugs)
- ☐ Violence
- ☐ Other: ______________

What do you think are the three most important social / environmental issues effecting the health of your community? (Choose 3)

- ☐ Access to doctor's office
- ☐ Limited access to healthy food
- ☐ School dropout rates / week educational system
- ☐ Lack of job opportunities
- ☐ Limited places to exercise
- ☐ Neighborhood Safety/Violence
- ☐ Lack of public parks and community entertainment centers
- ☐ Lack of affordable child care
- ☐ Child abuse/neglect
- ☐ Affordable housing
- ☐ Poverty
- ☐ Access to transportation
- ☐ Domestic violence
- ☐ Lack of mental health clinics
- ☐ Clean environment
- ☐ Race/Ethnicity discrimination
- ☐ Living near contaminating industry
- ☐ Other: ________________________

7.1.2 Tell us a bit about yourself:

How would you describe your health in general?

- ☐ Very good
- ☐ Good
- ☐ Not so good
- ☐ Not good at all
- ☐ Unknown / Refuse to share

Your age:

- ☐ 18-29
- ☐ 30-39
- ☐ 40-49
- ☐ 50-64
- ☐ 65-74
- ☐ +75

Your gender identity:

- ☐ Male
- ☐ Female
- ☐ Other:_________

How many children do you have? ___________________

Where were you born:

- ☐ Israel
- ☐ Other: ____________

If you were not born in Israel, what year did you move to Israel?__________

What is the highest level of education you have completed?

- ☐ High school diploma
- ☐ High school Matriculation diploma
- ☐ Professional school
- ☐ B.A.
- ☐ M.A. (including M.D)
- ☐ Ph.D.
- ☐ None of the above

Your religion:

- ☐ Jewish
- ☐ Christian
- ☐ Muslim
- ☐ Druze
- ☐ Other: ___________

Do you consider yourself:

- ☐ Very religious
- ☐ Religious
- ☐ Not so religious
- ☐ Not religious
- ☐ Unknown / Refuse to share

Marital status:

- ☐ Single
- ☐ Married
- ☐ Divorced
- ☐ Widow
- ☐ Single parent

Household Monthly Income

- ☐ Less than 2500 NIS
- ☐ 2,501-4,000 NIS
- ☐ 4,001-5,000 NIS
- ☐ 5,001-6,500 NIS
- ☐ 6,501 – 8,000 NIS
- ☐ 8,001 - 10,000 NIS
- ☐ 10,001 – 13,000 NIS
- ☐ 13,001 – 17,000 NIS
- ☐ 17,001 – 24,000 NIS
- ☐ Over 24,001 NIS

Perceived health status

- ☐ Not good
- ☐ Good
- ☐ Very good
