# Supplementary material for: Shaping health: conducting a community health needs assessment in culturally diverse peripheral population groups
Source: Int J Equity Health. 2022 Sep 12;21:131. doi: 10.1186/s12939-022-01735-z (PMC9469555; doi:10.1186/s12939-022-01735-z)
Supplement: Supplementary file 2 — Additional file 2: Table 1s. Adjusted odds ratios from multivariate logistic regression models predicting perceived community strengths among respondents (N = 759), Galilee residents Israel. Table 2s. Adjusted odds ratios from multivariate logistic regression models predicting perceived health needs among respondents (N = 759), Galilee residents Israel. Table 3s. Adjusted odds ratios from multivariate logistic regression models predicting perceived health problems among respondents (N = 759), Galilee residents Israel. Table 4s. Adjusted odds ratios from multivariate logistic regression models predicting social and structural determinants of health among respondents (N = 759), Galilee residents Israel. Table 4.1s. Adjusted odds ratios from multivariate logistic regression models predicting social and structural determinants of health among respondents (N = 759), Galilee residents Israel. [file 12939_2022_1735_MOESM2_ESM.pdf]

Supplementary file 2: Table 1s – Table 4.1s

Table 1s adjusted odds ratios from multivariate logistic regression models predicting perceived community strengths among respondents (N = 759), Galilee residents Israel

	Community services	Quality of life	Sense of community
Ethnicity (Ref: Arabs)	1.16	2.55**	3.01**
Female (Ref: male)	1.08	1.21	0.79
Age (ref: 18-29)			
30-39	5.24	0.94	1.05
40-49	10.37	1.57	0.61
50-64	6.76	1.82	0.54
65-74	7.48	0.96	1.33
75+	13.29	0.96	0.30
Education (Ref: high School)			
Professional school	0.89	0.96	2.13*
BA	0.71	1.37	2.96**
MA and above	0.87	1.36	4.23**
Municipal clusters (Ref: Eastern Galilee)			
Western Galilee – Beit HaKerem	0.97	1.29	1.07
Galil Amakim - Kineret Amakim	0.79	0.90	0.64
Locality (Ref: City)	1.73	1.05	3.40**
Religiosity (Ref: not religious)			
Not so religious	0.93	0.98	0.99
Religious	0.27**	0.88	1.98
Children number	0.98	0.94	1.13
Income	0.94	0.91	1.10

**P<0.0012 Bonferroni correction

Table 2s adjusted odds ratios from multivariate logistic regression models predicting perceived health needs among respondents (N = 759), Galilee residents Israel

	Health promotion	Hospitals	Community mental health services	Emergency services	Childcare	Elderly services	Community health services	Access to specialists
Ethnicity (Ref: Arabs)	0.43	0.50	4.56	2.62	0.95	1.56	1.02	1.04
Female (Ref: male)	0.78	1.53	2.18	0.48	0.97	1.64	0.83	0.95
Age (ref: 18-29)								
30-39	1.09	2.18	1.25	1.18	2.42	0.78	1.40	0.88
40-49	1.01	3.44	1.16	1.12	1.50	0.64	1.06	0.67
50-64	1.33	2.22	1.90	0.83	2.51	0.79	1.28	0.71
65-74	1.40	2.58	2.88	1.03	1.20	0.52	0.95	0.66
75+	0.76	4.95	-	3.14	4.2	0.46	1.68	1.44
Education (Ref: high School)								
Professional school	1.22	1.18	2.32	0.39	0.93	0.59	0.78	0.74
BA	1.03	1.16	3.66	0.43	1.73	1.12	1.06	0.97
MA and above	0.89	1.18	2.92	0.43	1.49	1.28	0.99	0.75
Municipal clusters (Ref: Eastern Galilee)								
Western Galilee – Beit HaKerem	1.99	0.81	1.34	1.05	0.86	0.68	0.98	1.20
Galil Amakim – Kineret Amakim	1.18	0.23	1.76	1.16	1.62	1.11	1.07	1.36
Locality (Ref: City)	0.92	0.89	0.95	0.49	0.78	0.86	1.32	0.78
Religiosity (Ref: not religious)								
Not so religious	0.52	1.45	1.06	1.35	0.70	0.99	1.32	1.07
Religious	0.90	1.56	2.05	1.31	0.85	0.93	1.62	1.10
Children number	0.94	0.84	0.87	0.97	0.99	1.01	1.02	1.08
Income	1.11	1.01	0.98	1.12	0.97	1.03	0.96	0.98

**P<0.0012 Bonferroni correction

Table 3s adjusted odds ratios from multivariate logistic regression models predicting perceived health problems among respondents (N = 759), Galilee residents Israel

	Age-related illness	Cancer	Dental problems	Diabetes	Heart disease and stroke	Infectious diseases	Lung disease (COPD)	Mental Health	Mother and Infant Health	Motor and Vehicle Crash	Obesity	Smoking	Substance Abuse	Violence
Ethnicity (Ref: Arabs)	4.44**	0.59	1.94	0.17**	0.59	9.80	1.30	2.47	9.29**	0.48	0.37**	0.41	1.47	0.07**
Female (Ref: male)	0.68	1.32	1.01	1.13	1.76	1.40	1.15	0.46	1.03	1.37	0.67	0.80	1.04	0.81
Age (ref: 18-29)														
30-39	0.64	1.13	1.52	1.43	1.24	0.89	1.06	1.09	1.05	0.36	1.42	0.70	0.45	2.24
40-49	0.71	1.32	1.17	3.29	1.60	1.00	1.3	0.76	0.55	0.31	1.55	0.50	0.29	0.97
50-64	0.81	1.33	1.09	2.51	2.38	0.45	0.95	0.61	0.34	0.30	3.96	1.01	0.42	2.56
65-74	2.95	1.82	1.31	3.55	1.96	0.23	0.73	0.44	0.26	0.04	2.23	0.49	0.68	1.80
75+	2.11	2.02	2.77	1.97	1.31	-	1.76	0.22	0.06	-	2.85	0.80	-	-
Education (Ref: high School)		1.46												
Professional school	1.11		0.89	0.85	0.52	0.60	1.80	0.83	1.13	0.95	2.33	2.08	1.30	-
BA	1.16	1.13	0.68	0.80	0.88	2.72	1.01	0.94	1.28	0.87	1.40	1.34	0.76	1.61
MA and above	1.25	1.10	1.0	0.71	0.64	2.85	1.09	1.11	0.91	0.98	1.36	1.28	1.06	2.79
Municipal clusters (Ref: Eastern Galilee)														
Western Galilee – Beit HaKerem	0.99	0.88	0.66	1.25	0.43**	0.44	1.40	1.36	0.73	2.44	1.33	0.77	6.07**	-
Galil Amakim – Kineret Amakim	1.09	072	0.89	0.52	0.69	0.52	0.33	1.50	0.93	1.66	1.71	2.06	5.09*	4.24
Locality (Ref: City)	1.24	1.38	0.82	0.85	0.58	3.14	0.89	0.54	2.05	1.15	0.57	0.58	0.49	0.69
Religiosity (Ref: not religious)														
Not so religious	0.93	1.08	1.12	0.86	0.92	0.64	2.62	0.54	0.82	1.43	1.03	0.43	2.53	1.5
Religious	0.90	0.66	1.14	1.03	1.03	1.49	1.32	0.42	1.16	0.92	1.17	1.11	3.32*	0.59
Children number	0.94	0.98	1.05	0.99	0.93	1.16	0.87	1.10	1.15	1.08	1.13	0.72	0.89	0.73
Income	1.05	1.05	0.87	1.04	1.01	0.89	1.01	0.87	1.06	0.95	0.98	1.04	1.05	1.06

Sexual Transmitted Infections was excluded because of low responses. **P<0.0012 Bonferroni correction

Table 4s adjusted odds ratios from multivariate logistic regression models predicting social and structural determinants of health among respondents (N = 759), Galilee residents Israel

	Residence near a polluting factory	Ethnicity discrimination	Pollution	Access to mental health services	Domestic violence	Access to transportation	Poverty	Affordable housing	Child abuse	Affordable childcare
Ethnicity (Ref: Arabs)	0.59	0.07**	0.74	5.95**	0.05**	23.5**	0.77	0.25	0.04**	0.31**
Female (Ref: male)	0.98	3.94	0.86	0.68	0.68	1.04	2.69	1.33	4.16	0.95
Age (ref: 18-29)										
30-39	1.60	0.08	1.14	0.74	0.84	0.62	1.43	1.09	--	1.11
40-49	2.12	0.30	0.51	0.91	0.20	0.79	1.02	0.77	0.25	0.94
50-64	2.01	0.18	0.49	0.59	1.27	0.77	1.06	0.45	0.84	0.73
65-74	3.24	0.11	0.97	0.55	4.55	0.90	--	1.01	0.71	1.14
75+	--	--	--	0.16	--	0.78	--	3.66	--	0.91
Education (Ref: high School)										
Professional school	--	--	2.48	0.56	--	0.83	1.03	0.43	0.82	0.83
BA	--	--	1.42	1.01	0.77	0.95	1.95	0.49	1.40	0.70
MA and above	--	--	1.83	1.03	0.09	1.87	1.63	0.33	0.47	0.48
Municipal clusters (Ref: Eastern Galilee)										
Western Galilee – Beit HaKerem	9.5**	--	3.47	0.61	0.70	1.29	0.64	3.08	0.38	1.32
Galil Amakim – Kineret Amakim	2.6	0.24	3.72	0.67	0.95	0.78	0.58	1.29	0.38	1.16
Locality (Ref: City)	0.57	1.69	1.84	1.56	0.51	4.07**	0.21	0.68	0.79	1.01
Religiosity (Ref: not religious)										
Not so religious	2.32	1.27	1.22	1.14	1.22	0.98	1.71	0.80	0.23	0.71
Religious	0.63	2.25	0.58	1.11	2.53	0.73	1.81	1.57	0.36	0.74
Children number	0.89	0.84	1.05	1.10	0.86	0.91	0.92	1.13	0.79	1.28
Income	1.20	0.95	0.83	1.08	1.15	1.02	0.78	1.11	0.88	1.09

**P<0.0012 Bonferroni correction

Table 4.1s adjusted odds ratios from multivariate logistic regression models predicting social and structural determinants of health among respondents (N = 759), Galilee residents Israel

	Affordable childcare	Parks and recreation	Neighborhood violence	Limited places to exercise	Lack of job opportunities	School dropout	Limited access to healthy food	Access to a doctor's office
Ethnicity (Ref: Arabs)	0.31**	0.10**	0.19**	0.32**	5.68**	0.14**	0.24**	24.6**
Female (Ref: male)	0.95	0.53*	1.08	0.83	1.34	0.46	0.77	1.56
Age (ref: 18-29)								
30-39	1.11	1.58	0.29	2.53	1.29	0.57	1.94	0.73
40-49	0.94	1.31	0.36	2.9	0.79	0.91	1.45	0.91
50-64	0.73	0.67	0.25	3.23	1.06	0.48	3.19	0.67
65-74	1.14	1.43	0.15	2.71	0.86	0.64	2.55	0.42
75+	0.91	--	--	3.09	0.40	--	--	0.42
Education (Ref: high School)								
Professional school	0.83	0.82	4.31	0.72	1.32	1.79	1.12	0.68
BA	0.70	0.74	1.67	1.09	0.96	0.86	0.72	0.95
MA and above	0.48	0.71	2.48	1.12	1.06	0.63	0.30	1.05
Municipal clusters (Ref: Eastern Galilee)								
Western Galilee – Beit HaKerem	1.32	0.61	4.288	0.55	0.59	2.32	1.54	0.52
Galil Amakim – Kineret Amakim	1.16	1.25	24.9**	0.65	0.58	0.96	0.76	0.54
Locality (Ref: City)	1.01	0.36**	0.23**	1.29	0.55	0.29	0.42	2.20**
Religiosity (Ref: not religious)								
Not so religious	0.71	0.60	0.68	1.08	0.83	1.16	0.61	1.30
Religious	0.74	1.21	1.44	1.80	0.58	0.70	1.02	1.03
Children number	1.28	1.07	1.06	0.94	0.92	1.13	1.02	1.04
Income	1.09	1.03	0.77	1.01	0.89	0.96	0.92	1.11

**P<0.0012 Bonferroni correction
